# Design of a Clip-On Modular Tactile Sensing Attachment Based on Fiber Bragg Gratings: Theoretical Modeling and Experimental Validation

**DOI:** 10.3390/s25195943

**Published:** 2025-09-23

**Authors:** Fengzhi Zhao, Yan Feng, Min Xu, Yaxi Li, Hua Zhang

**Affiliations:** 1 Robotics Institute, School of Mechanical and Automotive Engineering, Shanghai University of Engineering Science (SUES), Shanghai 201620, China; derrickfengzhizhao@163.com (F.Z.); m315124345@sues.edu.cn (Y.L.); huazhang@yeah.net (H.Z.); 2 Shanghai Collaborative Innovation Center for Robot Technology in Large-Component Intelligent Manufacturing, Shanghai 201620, China; 3 School of Information Engineering, Shanghai Zhongqiao Vocational and Technical University, Shanghai 201514, China; mhb@shzq.edu.cn

**Keywords:** tactile sensor, modularity, optical fiber Bragg grating, parametric opto-mechanical modeling, force perception

## Abstract

Despite widespread modular tooling in robots and automated systems, tactile sensing lags behind, constrained by custom and non-interchangeable sensors. To close this gap, we developed a clip-on cylindrical tactile module that combines a snap-fit Clip-on Cap (CC) with a plug-in Sensor Core (PSC) hosting an array of force sensing and temperature-reference fiber Bragg gratings (FBGs). An opto-mechanical model relates Bragg wavelength shifts to external forces through parameterized dimensions and remains applicable across varied module sizes. Two loading configurations are examined: Case I, a PSC fitted with a compliant PSC-solid insert, and Case II, a hollow PSC. Experiments across both configurations validate the model, with prediction errors below 8%. Case II offers up to twice the force sensitivity of Case I, whereas Case I maintains slightly higher linearity (R^2^ > 0.95). We propose a metric, Q, for assessing the trade-off among sensitivity, linearity, and dynamic lag; analyses with this metric establish that softer solid inserts enhance tactile force perception. The CC–PSC pair can be rapidly swapped or detached to meet diverse application needs. These results provide a transferable design and modeling framework for equipping robots—or other automated systems—with universally deployable, clip-on tactile perception.

## 1. Introduction

The pursuit of robot generality, namely, the ability of a single platform to tackle diverse tasks across unstructured settings, has become a defining goal for the next generation of field, medical, and collaborative robots [1,2]. Modularity implemented through plug-and-play sub-assemblies is widely regarded as the most pragmatic hardware route to that goal, because the ability to detach one sensing or actuation module and snap in another within seconds instantly reconfigures the robot’s functional repertoire without mechanical redesign or controller retraining.

Recent research on modular-reconfigurable robotics shows that such architectures deliver high task versatility and, by allowing damaged or task-specific modules to be swapped rather than overhauling an integrated platform, markedly shorten maintenance downtime in unstructured environments [3,4]. This function-oriented oriented modular and plug-and-play philosophy has long been embraced by the industrial robotics community. Industrial ecosystems already reflect this view: UR+ tool changers, OnRobot’s One System grippers, and FANUC CRX quick-release wrists all enable users to replace end effectors in under one minute, maintaining throughput as process recipes change [5]. Yet most of these modular efforts—both academic and industrial—concentrate on chassis, joints, or actuation, whereas a function-level module for tactile perception remains conspicuously underdeveloped [6,7]. As a result, today’s robots can swap a gripper in seconds, but still require bespoke, permanently bonded tactile sensors, leaving force perception the slowest and least flexible element in an otherwise plug-and-play ecosystem.

Advances in three aspects—flexible micro-engineered e-skins, vision-based tactile sensors (VTS), and optical-fiber devices—have recently boosted tactile sensing by raising sensitivity, bandwidth, and multimodal capability. Micro-textured e-skins (pyramids, porous, and biomimetic surfaces) extend linear range and sensitivity, while reviews still flag integration and scalability as open challenges [8,9]. Materials-centered surveys echo this, citing porous graphene skins that deliver kPa-to-MPa operation windows and rapid response [10,11]. In parallel, Wang et al. report a hydrogel-based flexible patch integrating proximity, pressure, and temperature sensing on soft substrates, exemplifying multimodal e-skin implementations [12]. Yet these laminates are usually permanently bonded to a substrate; most cannot be detached or resized in the field, and some glove-style versions are explicitly single-use [13,14,15]. VTSs provide rich contact imagery and multi-axis force inference, with steadily maturing modeling and calibration pipelines [16,17,18]. Classic GelSight-type stacks—coating, elastomer, optics/LEDs, camera—integrate well into fingers and palms but form a single replace-as-one block; precision often demands full recalibration, and camera heating can shift elastomer properties [19,20]. Thus, VTSs rarely meet the tool-change timescale expected of hot-swappable modules. Optical-fiber tactile approaches, especially FBG arrays, add EMI immunity, compact wiring, and multiplexed distributed sensing, achieving sub-millinewton resolution with fast response [21,22,23]. However, temperature drift usually necessitates compensation via a separate reference grating—an aspect many prototypes omit [24]. Even “modular” FBG pads embed the fiber mesh in multilayer laminates, so resizing or repair still means re-molding and recalibration rather than a simple clip-off swap.

Collectively, the literature confirms strides in sensing fidelity and function, yet most tactile layers remain “bond-once-use-forever” components: they are integrated as permanent stacks or laminates, with few examples that can be clipped on, unplugged, or resized within minutes without destructive removal and fresh calibration. This directly motivates the present work’s focus on a plug-and-play, detachable tactile module.

Nevertheless, among the limited prototypes that attempt to meet this requirement, the geometric design space explored thus far remains narrowly confined. To date, the majority of tactile units that exhibit or are explicitly designed for modular, plug-and-play potential have been realized as planar skins, dome-shaped pads, or gripper-palm inserts [25,26,27,28,29]. In contrast, comparatively little attention has been devoted to cylindrical, tip-mounted modules that conform to the rounded geometry of human fingertips and many industrial tool tips. Such a form factor is increasingly relevant to collaborative manipulation tasks—where contact is localized at the distal end and the available mounting footprint is inherently circular, reducing to a single point contact [30,31,32]. Consequently, designing a clip-on, parametrically scalable cylindrical tactile module and formulating its underlying sensing model constitutes both a critical requirement and a formidable challenge, addressing an application niche—cylindrical, tip-mounted fingertips—common in collaborative manipulation yet largely overlooked in existing research.

Motivated by these deficiencies, this work bridges the gap through the following approaches:

1. Introducing a clip-on, parametrically scalable cylindrical tactile module. A two-piece architecture—snap-fit cap and plug-in FBG sensor core—enables attachment, detachment, or resizing within seconds, supporting plug-and-play replacement.
2. Developing a parametric, closed-form opto-mechanical model. A geometry-aware opto-mechanical formulation links the module’s principal dimensions (cap aperture and core wall thickness) to its wavelength-to-force coefficient, providing a rapid, script-based route to generate diameters from 10 mm to 22 mm without re-molding.
3. Establishing and validating a dual sensing model, together with a quantitative performance metric. Separate analytical formulations are derived for (i) the clip-on module equipped with a Plug-in Sensor Core (PSC) and (ii) the module operated without the PSC-solid insert. Experiments conducted across different cases validate the sensing model and yield metric *Q* for assessing tactile force perception.

## 2. Clip-On Tactile Module: Design and Fabrication

### 2.1. Mechanical Architecture of the Clip-On Cap and Plug-In Sensor Core

**Figure 1:**
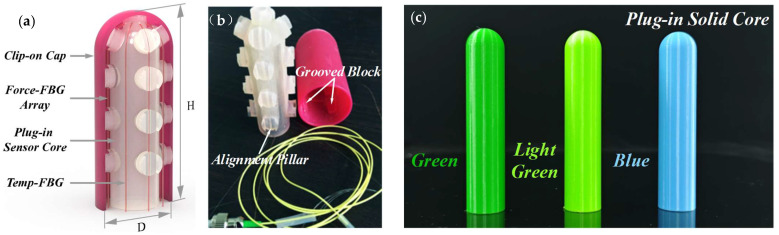
Clip-on tactile sensor module: (**a**) cross-sectional view showing the Clip-on Cap (CC), Plug-in Sensor Core (PSC), Force-FBG array (×4), and Temp-FBG (reference); (**b**) prototype of the assembled CC-PSC module with embedded FBGs; and (**c**) color-coded Plug-in Solid Cores (PSC-solid) used for mechanical comparison.

The clip-on tactile module is intended for mounting on a human or robotic fingertip to evaluate force sensing. Figure 1 presents a completed prototype with a cross-sectional view. The module consists of two detachable parts: a Plug-in Sensor Core (PSC) and a Clip-on Cap (CC) (Figure 1a). Both components are hollow cylinders terminated by a spherical dome. Four grooved pillars on the PSC mate precisely with complementary alignment pillars on the inner surface of the CC (Figure 1b). Each grooved pillar contains a 1 mm through-hole that guides the optical fibers. The module’s outer diameter D is 34 mm, its overall height H is 77 mm, and the wall thicknesses of both PSC and CC are 1.5 mm. To emulate a fingertip during testing, a Plug-in Solid Core (PSC-solid)—a solid cylinder with a matching spherical top—was fabricated as the loading indenter during mechanical tests.

### 2.2. Additive Manufacturing and Assembly Procedure

PolyJet printing is an additive manufacturing process with the capability to make parts, prototypes, and models in multiple materials, colors, and color textures. The Stratasys J750 printer has six jetting heads, one for spraying the support material, and the other five for spraying five different colors of ink. The liquid photopolymers as ink are jetted and cured simultaneously by a UV lamp within the printer, creating a solid model that is precise and accurate. After printing, the gel-like support material in through-holes and substance is easily washed away. In this work, rubber-like material and Digital ABS were used as photopolymer inks. Two Plug-in Sensor Cores (PSCs) were printed by PolyJet on the Stratasys J750 and are hereafter referred to as 1^#^ PSC and 2^#^ PSC. Three Plug-in Solid Cores (PSC-solid) were likewise printed in different materials and colors to represent the varied sensing requirements encountered in diverse application scenarios, as shown in Figure 1c.

The parts were PolyJet-printed using rubber-like photopolymer and Digital ABS, as described above. For modeling and analysis, the effective Young’s moduli used are as follows: Plug-in Sensor Core (PSC) *E*_1_ = 3.2 × 10^6^ Pa in Case I and 1.4 × 10^6^ Pa in Case II; Clip-on Cap (CC) *E*_2_ = 44 × 10^6^ Pa; PSC-solid inserts *E*_S_: blue *E*_S-B_ = 2.0 × 10^9^ Pa, green *E*_S-G_ = 7.2 × 10^8^ Pa, and light-green *E*_S-LG_ = 3.0 × 10^7^ Pa.

### 2.3. FBG Array Layout and Attachment Strategy

**Figure 2:**
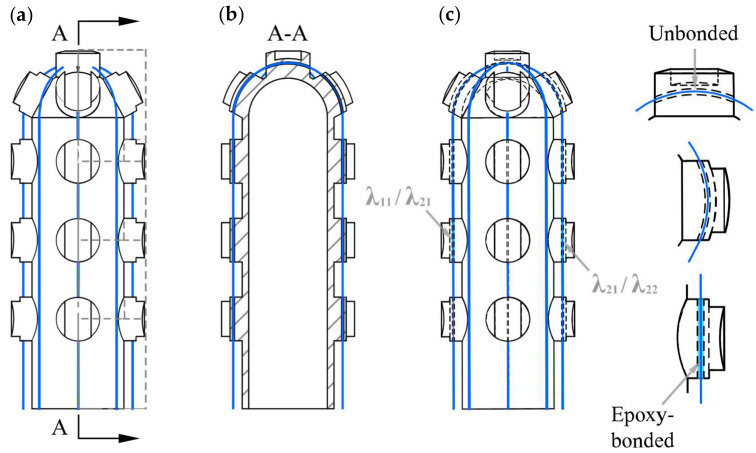
Placement and attachment of FBGs in the clip-on tactile sensor module: (**a**) external view of the Plug-in Sensor Core (PSC) showing the routing of the Force-FBG array; (**b**) longitudinal section A–A illustrating the axial grooves that host the four Force-FBGs; (**c**) comparison of unbonded and epoxy-bonded attachment configurations.

After being written into Bragg gratings, the FBGs are recoated with acrylate coating. We use *λ*_ij_ to name the FBGs, subscript letter i (i = 1,2) indicates the PSC index, and j (j = 1, 2) indicates the location. The FBG array written into two Bragg gratings with two different initial wavelengths λ_11_ and λ_12_ is embedded into 1^#^ PSC, and the other FBG array, with two different initial wavelengths, λ_21_ and λ_22_, is embedded into 2^#^ PSC, as shown in Figure 2a. In addition, an unbonded FBG for temperature compensation—designated Temp-FBG—was routed through the top hole and left free-hanging inside each PSC, as shown in Figure 1a and Figure 2. The length of the Bragg gratings is 5 mm. The distance between the two Bragg gratings matches the spacing distance between adjacent grooved pillars on the PSC. We threaded the FBG arrays through the holes in the pillars and epoxy-bonded at one end, ensuring that the FBGs deform synchronously with the PSC (Figure 2b).

## 3. Experimental Evaluation

**Figure 3:**
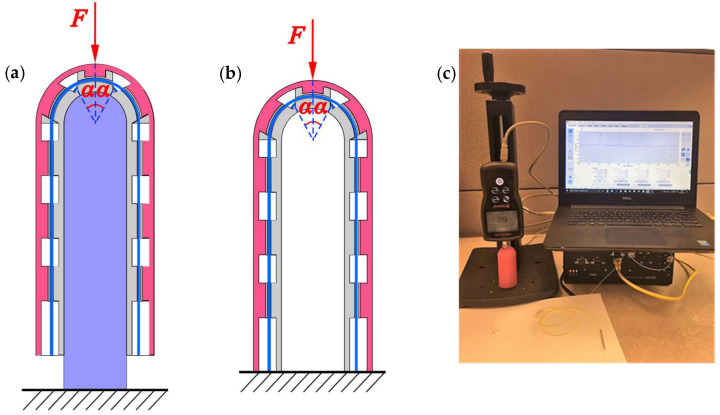
Tactile force sensing experiment: (**a**) schematic of Case I; (**b**) schematic of Case II; (**c**) experimental setup.

The initial wavelengths of the FBGs were λ_11_ = 1546.295 nm, λ_12_ = 1524.570 nm, λ_21_ = 1540.060 nm, λ_22_ = 1530.701 nm, and λ_TEMP_ = 1560.146 nm, measured at a room temperature of 23 °C. The clip-on tactile modules’ responses to impulse signals were tested. The tactile sensing experiment is divided into two cases: Case I, 1^#^ PSC is equipped with a PSC-solid insert and the assembly is clamped to the test table (Figure 3a); Case II, 2^#^ PSC with no solid insert, is clamped to the table (Figure 3b). We divided the experiment into two groups, and Table 1 shows the details. We employed a force test stand (SHPMT S209, Shimano, Peterborough, ON, Canada) to push the module and optical sensing instrument (SM125, Micron Optics, Atlanta, GA, USA, Acquisition rate is 2 Hz, Resolution is 1 pm) to assess the module’s contact force tactile sensing, shown in Figure 3c. We recorded the force data and the wavelength data simultaneously and obtained the FBGs’ sensitivity *K*, linear correlation coefficient *R*^2^, and the maximum time lag Δ*t*_max_ between the force peak and the transmission wavelength peak for kinematic analysis. To provide a comprehensive measure of tactile sensing performance, this paper introduces a metric *Q* = ∣*K*∣ * *R*^2^∕Δ*t*_max_ for assessing the module’s tactile force perception. The larger the value of Q, the better the tactile force sensing.

### 3.1. Tactile Sensing Results of 1^#^ PSC

**Figure 4:**
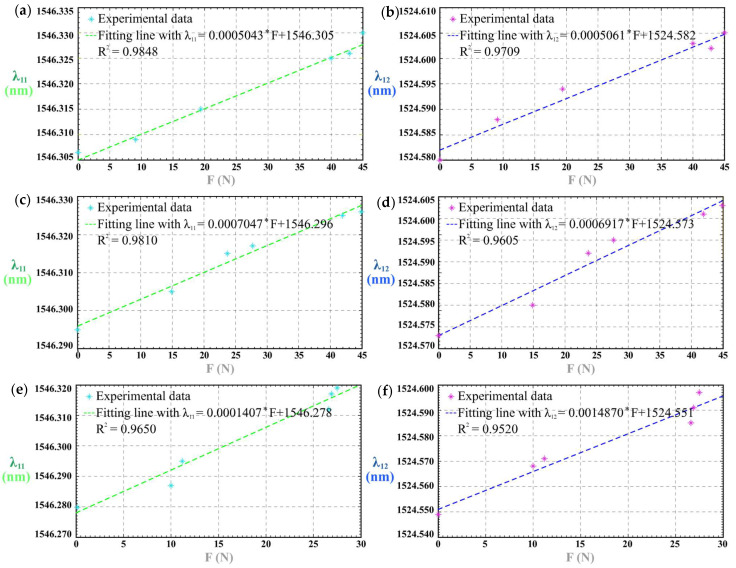
1^#^ PSC’s responses to the impulse loads: (**a**) sensitivity of λ_11_ of 1^#^-I-B; (**b**) sensitivity of *λ*_12_ of 1^#^-I-B; (**c**) spectra of 1^#^-I-G; (**d**) sensitivity of *λ*_11_ of 1^#^-I-G; (**e**) sensitivity of *λ*_12_ of 1^#^-I-G; (**f**) spectra of 1^#^-I-LG; (**g**) sensitivity of λ_11_ of 1^#^-I-LG; (**h**) sensitivity of λ_12_ of 1^#^-I-LG.

**Figure A1:**
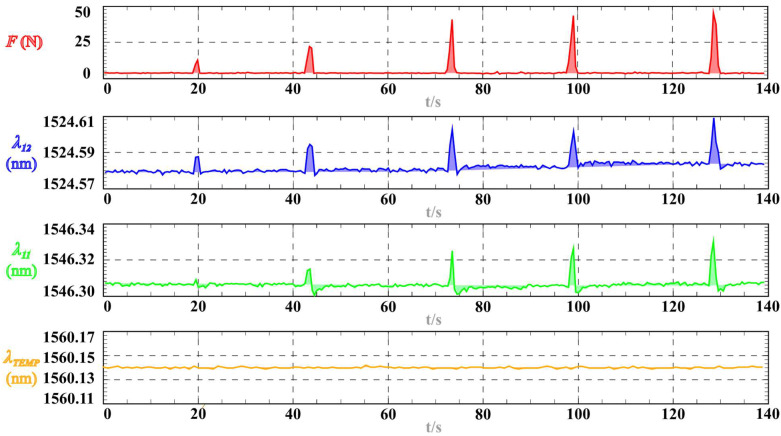
Bragg wavelength shift (Δ*λ*) versus applied force (*F*)—Case I.

**Figure A2:**
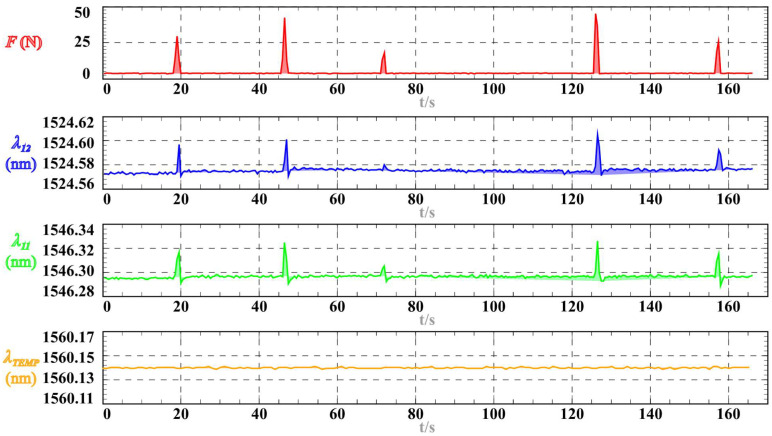
Bragg wavelength shift (Δ*λ*) versus applied force (*F*)—Case I.

**Figure A3:**
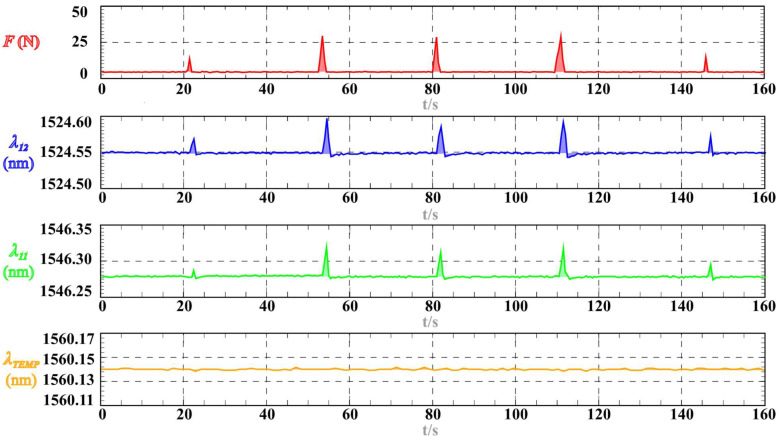
Bragg wavelength shift (Δ*λ*) versus applied force (F)—Case I.

With the blue PSC-solid insert, PSC-1 responded to impulse loads as illustrated in Figure A1 and Figure 4a,b. Using the green PSC-solid insert, the responses are shown in Figure A2 and Figure 4c,d; with the light-green insert, they are presented in Figure A3 and Figure 4e,f. In every case, the Bragg wavelength increased monotonically with external force. We use *K*_11-I-B_ as the sensitivity of tactile force for *λ*_11_ of Group 1^#^-I-B, *K*_12-I-B_ as the sensitivity of tactile force for *λ*_12_ of Group 1^#^-I-B, *K*_11-I-G_ for *λ*_11_ of Group 1^#^-I-G, *K*_12-I-G_ for *λ*_12_ of Group 1^#^-I-G, and *K*_11-I-LG_ for *λ*_11_ of Group 1^#^-I-LG, and *K*_12-I-LG_ for *λ*_12_ of Group 1^#^-I-LG. Table 2 shows the detailed results of sensitivity, linear correlation coefficient *R*^2^ and the maximum time lag Δ*t*_max_. In all analyses, the force sensitivity is defined as *K* = Δ*λ*/*F* (pm/N), i.e., the slope of the linear fit between the Bragg wavelength shift and the applied force. It can be concluded from the results that sensitivity increases as the Young’s modulus of the PSC-solid insert decreases.

1^#^ PSC shows different impulse-load responses with the blue/green/light-green PSC-solid inserts because these inserts have distinct Young’s moduli. In all three cases, the Bragg wavelength increases monotonically with force, but the measured sensitivity is higher for the lower-modulus insert (Figure 4, Table 2). This behavior accords with the model’s parametric analysis for Case I, which predicts a linear red shift with force and a decreasing sensitivity as ES increases.

### 3.2. Tactile Sensing Results of 2^#^ PSC

**Figure 5:**
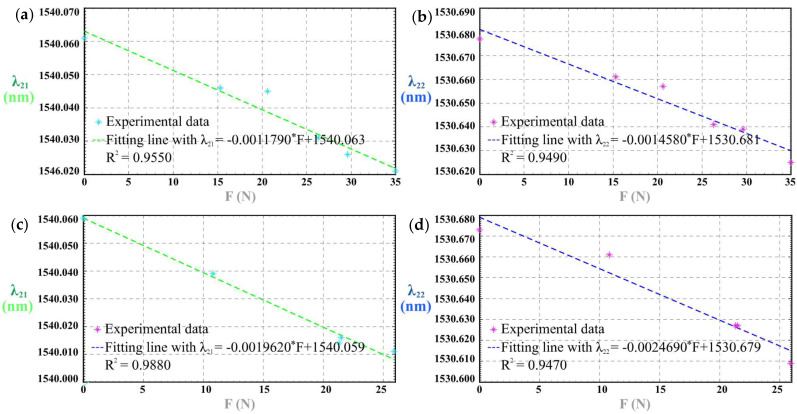
2^#^ PSC’s responses to the impulse loads: (**a**) sensitivity of λ_22_ of 2^#^-II-1; (**b**) spectra of 2^#^-II-2; (**c**) sensitivity of λ_21_ of 2^#^-II-2; (**d**) sensitivity of λ_22_ of 2^#^-II-2.

Without any PSC-solid insert, 2^#^ PSC’s responses to the impulse loads are shown in Figure 5. The results indicate that the wavelength decreases as the external load increases. We use *K*_21-II-1_ as the sensitivity of tactile force for λ_21_ of Group 2^#^-II-1, *K*_22-II-1_ as the sensitivity of tactile force for λ_22_ of Group 2^#^-II-1, *K*_21-II-2_ as the sensitivity of tactile force for λ_21_ of Group 2^#^-II-2, and *K*_22-II-2_ as the sensitivity of tactile force for λ_22_ of Group 2^#^-II-2. Table 3 shows the detailed results of sensitivity, linear correlation coefficient R^2^ and the maximum time lag Δ*t*_max_.

We can obtain that the tactile sensitivity *K*_-I_ Case I is smaller than *K*_-II_ in Case II, whereas *R*^2^ in Case I is larger than *R*^2^ in Case II. We use the symbols $\bar{\Delta t_{I}}$ and $\bar{\Delta t_{II}}$ for the average time lag of Case I and Case II. From the experimental results, we obtain $\bar{\Delta t_{I}}=0.64$ s, $\bar{\Delta t_{II}}=0.88$ s.

## 4. Tactile Sensing Model and Theory

For a single-mode fiber (SMF) FBG, the Bragg wavelength shifts when it is under strain and temperature, with Δ*λ_B_* expressed as Equation (1a) [33,34,35]:

(1a) $$\Delta \lambda_{B}=2n\Lambda \left(\left\{1-\left(\frac{n^{2}}{2}\right)[p_{12}-\mu (p_{11}+p_{12})]\right\}\varepsilon_{z}+[\alpha +\frac{\left(\frac{dn}{dT}\right)}{n}]\Delta T\right)$$

where *n* is the effective index of the fiber core, *Λ* is the grating pitch, α is the coefficient of linear thermal expansion of the fiber, *p*_i,j_ are the Pockels coefficients of the stress-optic tensor, *μ* is Poisson’s ratio, Δ*T* is the temperature change, and *ε*_z_ is the applied axial strain. The factor *2nΛ* is the resonance condition of a Bragg grating and is expressed as *λ*_B_, the Bragg wavelength. The factor {(*n*^2^/2)[*p*_12_ − *μ* (*p*_11_ *+ p*_12_)]} is usually expressed as *p*_e_, the Pockels constant. The factor [(*dn*/*dT)*/*n*] is usually expressed as *ξ*, the thermo-optic coefficient. Equation (1a) can be given simply by Equation (1b) [33,34,35].

(1b) $$\Delta \lambda_{B}=\lambda_{B}(\alpha +\xi )\Delta T+\lambda_{B}(1-p_{e})\varepsilon_{z}$$

### 4.1. Opto-Mechanical Modeling of the Clip-On Module

**Figure 6:**
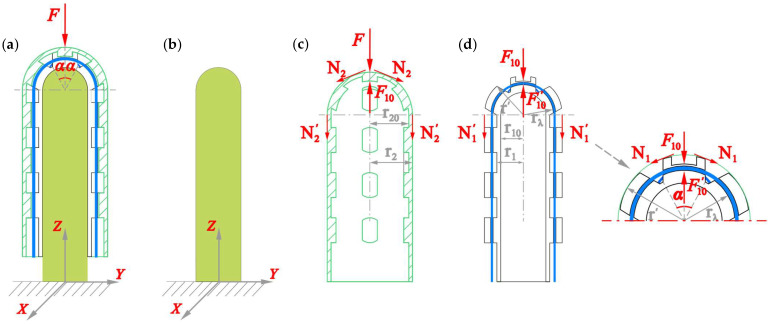
Schematic of the load on the clip-on tactile sensor module for Case I: (**a**) general deformation; (**b**) load on the PSC-solid; (**c**) stresses on the clip-on cap; (**d**) load on the spherical cap.

In this section, some important equations are summarized to describe the wavelength shifts in the FBGs. We used two elastomeric rotating shell models to assess the tactile module’s contact force sensing when pushing the module, including two cases: (I) equipped with a PSC-solid insert, and (II) without a PSC-solid insert. The spherical cap of the Clip-on Cap (CC) is subjected to the external force *F*, and this *F* induces the deformations of the PSC. The coordinate is shown in Figure 6a. We denote *F*_10_ as the load acting on the spherical cap of the CC. We let some symbols describe the mentioned parameters, and their descriptions are shown in Table 4. There are five assumptions made to simplify the model:

(1) All the material is isotropic, and the deformations are in the fully elastic phase.

(2) The external force *F* acts on the neutral surface of the spherical cap of the PSC.

(3) There is no sliding between the CC and the PSC, and they touch with each other tightly.

(4) There is no sliding between the PSC-solid and the PSC, and they touch with each other tightly.

(5) FBG sensors contact closely with the PSC and there is no relative displacement between the PSC and FBGs.

#### 4.1.1. Modeling of Case I

In this case, the clip-on tactile module is equipped with a PSC-solid insert inside, and its end is free. When the force *F* loads on the top of the Clip-on Cap (CC), the solid PSC-solid insert is pressed, whereas the clip-on tactile module will extend along the PSC-solid insert freely, as shown in Figure 6a,b. Figure 6d shows the schematic diagram of the stress loaded on the Plug-in Sensor Core (PSC)’s spherical cap. According to the theory of elasticity [36,37], we write Equation (2a) to solve the meridian stress *N*_1_:

(2a) $$\Delta N_{1}=\frac{F_{10}-F_{10}^{\prime }}{2\pi r^{\prime }{sin}^{2}\alpha }$$

when α = 90°, the tension stress *N*_1_′ on the cylinder section of the PSC can be expressed as Equation (2b):

(2b) $$N_{1}^{\prime }=\frac{F_{10}-F_{10}^{\prime }}{2\pi r^{\prime }}$$

when *r* = *r*_λ_, we can obtain the axial tension *f*_λ_ on the FBGs embedded in 1^#^ PSC, shown in Equation (3):

(3) $$f_{\lambda }=N_{1}^{\prime }\times r_{\lambda }=\frac{\left(F_{10}-F_{10}^{\prime }\right)r_{\lambda }}{2\pi r^{\prime }}$$

Substituting it into Equation (1b) and considering Δ*T* = 0, the Bragg wavelength shift Δ*λ*_I_ in Case I can be obtained as Equation (4).

(4) $${\Delta \lambda }_{I}=\lambda \left(1-p_{e}\right)\frac{\left(F_{10}-F_{10}^{\prime }\right)r_{\lambda }}{2\pi r^{\prime }r_{0}^{2}E_{0}}$$

Similarly to *N*_1_′, Equation (5) can be derived for the tension stress *N*_2_′on the cylinder section of the Clip-on Cap (CC).

(5) $$N_{2}^{\prime }=\frac{F-F_{10}}{2\pi r_{2}}$$

The stress and the strain of the cylinder section of the CC satisfies Equation (6):

(6) $$\left\{\begin{array}{c} \sigma_{2}=\frac{N_{2}^{\prime }\times r^{\prime }}{A_{2}}=-\frac{F-F_{10}}{2\pi^{2}\left({r_{2}}^{2}-{r_{20}}^{2}\right)}\\ \varepsilon_{2}=\frac{\sigma_{2}}{E_{2}}=-\frac{F-F_{10}}{2\pi^{2}\left({r_{2}}^{2}-{r_{20}}^{2}\right)E_{2}} \end{array}\right.$$

and the stress and the strain of the cylinder section of the Plug-in Sensor Core (PSC) satisfies Equation (7):

(7) $$\left\{\begin{array}{c} \sigma_{1}=\frac{N_{1}^{\prime }\times r^{\prime }}{A_{1}}=-\frac{F_{10}-F_{10}^{\prime }}{2\pi^{2}\left({r_{1}}^{2}-{r_{10}}^{2}\right)}\\ \varepsilon_{1}=\frac{\sigma_{1}}{E_{1}}=-\frac{F_{10}-F_{10}^{\prime }}{2\pi^{2}\left({r_{1}}^{2}-{r_{10}}^{2}\right)E_{1}} \end{array}\right.$$

Without any difficulty, we can obtain the strain of the PSC-solid insert as Equation (8):

(8) $$\varepsilon_{sz}=-\frac{F_{10}^{\prime }}{E_{s}A_{s}}=-\frac{F_{10}^{\prime }}{\pi {r_{10}}^{2}E_{s}}$$

Taking account of the assumptions in Section 4.1, the boundary condition satisfies Equation (9):

(9) $$\left\{\begin{array}{c} \varepsilon_{1}=-\varepsilon_{sz}\\ \varepsilon_{1}=\varepsilon_{2} \end{array}\right.$$

Accordingly, the Bragg wavelength shift Δλ_I_ in Equation (4) becomes the following:

(10) $${\Delta \lambda }_{I}=\lambda \left(1-p_{e}\right)\frac{Fr_{\lambda }}{\pi r^{\prime }r_{0}^{2}E_{0}}\frac{\left({r_{1}}^{2}-{r_{10}}^{2}\right)E_{1}}{2\pi \left({r_{1}}^{2}-{r_{10}}^{2}\right)E_{1}+2\pi \left({r_{2}}^{2}-{r_{20}}^{2}\right)E_{2}+{r_{10}}^{2}E_{s}}=K_{I}F$$

where

$$K_{I}=\lambda \left(1-p_{e}\right)\frac{r_{\lambda }}{\pi r^{\prime }r_{0}^{2}E_{0}}\frac{\left({r_{1}}^{2}-{r_{10}}^{2}\right)E_{1}}{2\pi \left({r_{1}}^{2}-{r_{10}}^{2}\right)E_{1}+2\pi \left({r_{2}}^{2}-{r_{20}}^{2}\right)E_{2}+{r_{10}}^{2}E_{s}}>0\;.$$

#### 4.1.2. Modeling of Case II

**Figure 7:**
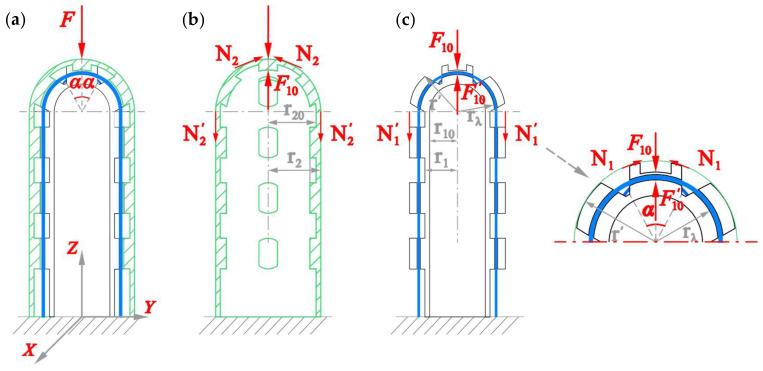
Schematic of the load on clip-on tactile sensor module for Case II: (**a**) general deformation; (**b**) load on the PSC; (**c**) stresses on the clip-on cap.

In Case II, the clip-on tactile module is on the table without any PSC-solid inside. When the force *F* loads on the spherical top of the Clip-on Cap, the Plug-in Sensor Core is pressed, shown in Figure 7.

According to the theory of elasticity [36,37], we write Equation (11) to solve the meridian stress *N*_1_:

(11) $$N_{1}=\frac{F_{10}}{2\pi r^{\prime }{sin}^{2}\alpha }$$

where *α* = 90°, the compression stress *N*_1_′ on the cylinder section of the Plug-in Sensor Core (PSC), can be expressed as Equation (12).

(12) $$N_{1}^{\prime }=-\frac{F_{10}}{2\pi r^{\prime }}$$

For FBGs embedded in 2^#^ PSC named as *λ*_21_ and *λ*_22_*,* the compression force *f’*_λ_ along their axial direction can be expressed as Equation (13):

(13) $$f_{\lambda }^{\prime }=-N_{1}^{\prime }\times r_{\lambda }=-\frac{F_{10}r_{\lambda }}{2\pi r^{\prime }}$$

Similarly to Equation (4), the Bragg wavelength shift Δ*λ*_II_ in Case II is expressed as Equation (14):

(14) $$\Delta \lambda_{II}=-\lambda \left(1-p_{e}\right)\frac{F_{10}r_{\lambda }}{2\pi {r_{0}}^{2}r^{\prime }E_{0}}$$

Next, we derive the relation between *F*_10_ and *F*. The meridian stress *N*_2_ can be expressed as Equation (15a). When *α* = 90°, the cylinder section of the Clip-on Cap is subjected to the compressive stress *N*_2_′, as shown in Equation (15b):

(15a) $$N_{2}=\frac{F-F_{10}}{2\pi r_{2}{sin}^{2}\alpha }$$

(15b) $$N_{2}^{\prime }=-\frac{F-F_{10}}{2\pi r_{2}}$$

The stress and the strain of the cylinder section of the CC σ_2_ satisfies Equation (16)

(16) $$\left\{\begin{array}{c} \sigma_{2}=\frac{N_{2}^{\prime }\times r_{20}}{A_{2}}=\frac{-(F-F_{10})\times r_{20}}{2\pi^{2}r_{2}({r_{2}}^{2}-{r_{20}}^{2})}\\ \varepsilon_{2}=\frac{\sigma_{2}}{E_{2}}=\frac{-(F-F_{10})\times r_{20}}{2\pi^{2}r_{2}({r_{2}}^{2}-{r_{20}}^{2})E_{2}} \end{array}\right.$$

Meanwhile, the stress and the strain of the cylinder section of the Plug-in Sensor Core (PSC) σ_1_ satisfies Equation (17):

(17) $$\left\{\begin{array}{c} \sigma_{1}=\frac{N_{1}^{\prime }\times r\prime }{A_{1}}=\frac{-F_{10}}{2\pi^{2}({r_{1}}^{2}-{r_{10}}^{2})}\\ \varepsilon_{1}=\frac{\sigma_{1}}{E_{1}}=\frac{-F_{10}}{2\pi^{2}({r_{1}}^{2}-{r_{10}}^{2})E_{1}} \end{array}\right.$$

There is no relative sliding and displacement between the CC and the PSC, and the boundary condition is as Equation (18):

(18) $$\varepsilon_{1}=\varepsilon_{2}$$

Solving Equations (16)–(18) for *F*_10_, we obtain the following:

(19) $$F_{10}=F\times \frac{E_{1}r_{20}({r_{1}}^{2}-{r_{10}}^{2})}{E_{2}r_{2}({r_{2}}^{2}-{r_{20}}^{2})+E_{1}r_{20}({r_{1}}^{2}-{r_{10}}^{2})}$$

Substituting Equation (19) into Equation (14), we therefore obtain Equation (20). From it, we know that the FBGs’ Bragg wavelength is a linear function of external force *F* and will be blue shifted with the increase in F in Case II:

(20) $$\Delta \lambda_{II}=-\lambda (1-p_{e})F\times \frac{E_{1}({r_{1}}^{2}-{r_{10}}^{2})}{E_{2}({r_{2}}^{2}-{r_{20}}^{2})+E_{1}({r_{1}}^{2}-{r_{10}}^{2})}\times \frac{r_{\lambda }}{2\pi^{2}{r_{0}}^{2}r^{\prime }E_{0}}=K_{II}F$$

where

$$K_{II}=-\lambda (1-p_{e})\frac{E_{1}({r_{1}}^{2}-{r_{10}}^{2})r_{\lambda }}{{[E}_{2}({r_{2}}^{2}-{r_{20}}^{2})+E_{1}({r_{1}}^{2}-{r_{10}}^{2})]2\pi^{2}{r_{0}}^{2}r^{\prime }E_{0}}<0\;.$$

### 4.2. Parametric Analysis

In this section, we analyze the theoretical tactile sensitivity and the effect of Young’s modulus on sensitivity. In what follows, *E*_1_ denotes the Young’s modulus of the PSC, *E*_2_ that of the CC, and *E*_S_ that of the PSC-solid insert (with *E*_S-B_, *E*_S-G_, *E*_S-LG_ corresponding to the blue, green, and light-green variants, respectively); the corresponding numerical values are provided in Table 5.

#### 4.2.1. Analysis of Case I

**Figure 8:**
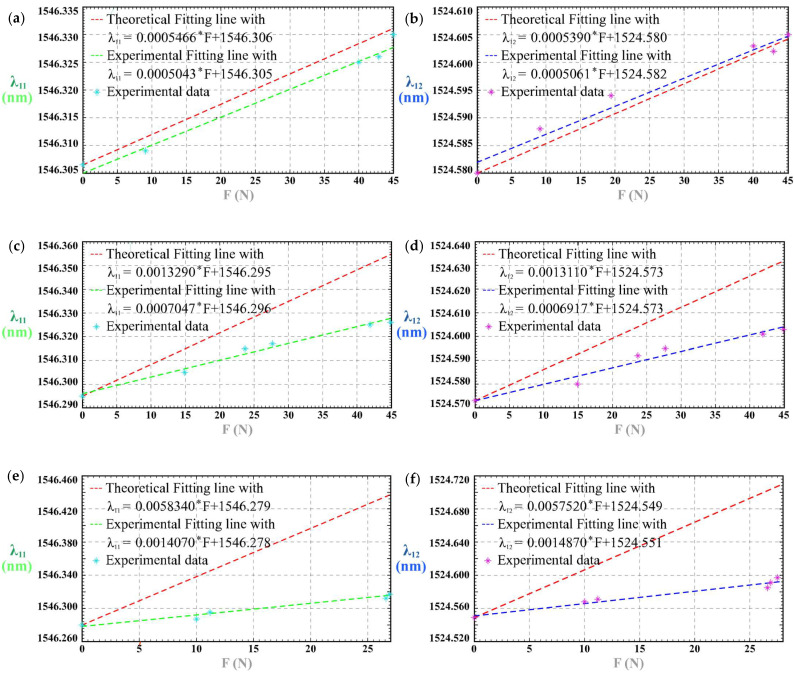
Comparison of the theoretical and the experimental sensitivity in Case I; (**a**) *λ*_11_ of 1^#^-I-B; (**b**) *λ*_12_ of 1^#^-I-B; (**c**) *λ*_11_ of 1^#^-I-G; (**d**) *λ*_12_ of 1^#^-I-G; (**e**) *λ*_11_ of 1^#^-I-LG; (**f**) *λ*_12_ of 1^#^-I-LG.

**Figure 9:**
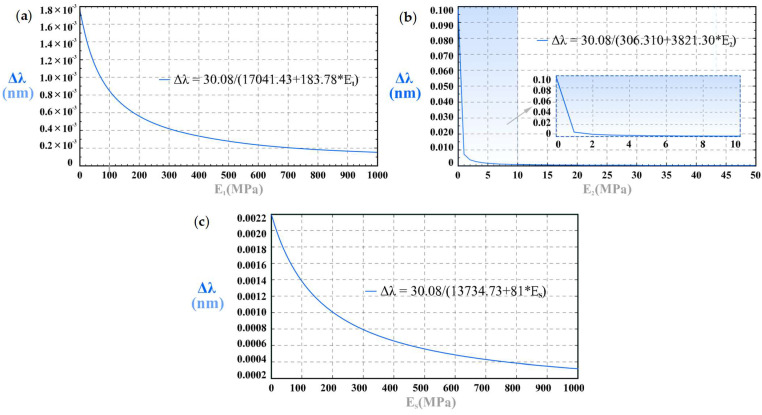
Effects of Young’s modulus on wavelength shift in Case I: (**a**) plug-in sensor core’s Young’s modulus *E*_1_; (**b**) clip-on cap’s Young’s modulus *E*_2_; (**c**) PSC-solid’s Young’s modulus *E*_s_.

As shown in Figure 8, the model in Equation (10) predicts that the FBGs’ Bragg wavelength is a linear function of external force F and will be red-shifted with the increase in F in Case I, which is in accord with the experimental results. As illustrated in Figure 9a,b, the model works well for the clip-on tactile module with the blue PSC-solid insert, showing that the relative errors between the theoretical sensitivity and the experimental sensitivity are 7.74% and 6.10%, respectively. With the decrease in the Young’s modulus of the PSC-solid insert, the relative error increases.

Figure 9a shows the effect of Young’s modulus *E*_1_ on the wavelength shift Δ*λ* when *F* = 50 N, *E*_2_ = 44 MPa, and *E*_s_ = 44 MPa. As the *E*_1_ increases, Δ*λ* decreases; that is, the tactile sensitivity decreases. When the *E*_1_ achieves 800 MPa, the tactile sensitivity gradually stabilizes and achieves a constant value. Figure 9b shows the relation of Δ*λ* and *E*_2_ when the external force *F* = 50 N, *E*_1_ = 1.4 MPa, and *E*_s_ = 44 MPa. With the increase of *E*_2_, the tactile sensitivity decreases sharply at first. When *E*_2_ increases to 5 MPa, the tactile sensitivity gradually becomes zero; that is, the tactile sensitivity does not fluctuate with the change of *E*_2_ while the other parameters remain unchanged. Figure 9c shows the relation between *F* = 50 N, *E*_1_ = 1.4 MPa, and *E*_2_ = 44 MPa. As the Es increases, the tactile sensitivity shows a downward trend, which agrees with the experimental results. When *E*_s_ ≤ 500 MPa, the tactile sensitivity decreases rapidly with the increase of *E*_s_. When *E*_s_ > 500 MPa, the tactile sensitivity decreases slowly and then tends to be constant gradually. The change of *E*_2_ has the greatest impact on tactile sensitivity.

#### 4.2.2. Analysis of Case II

**Figure 10:**
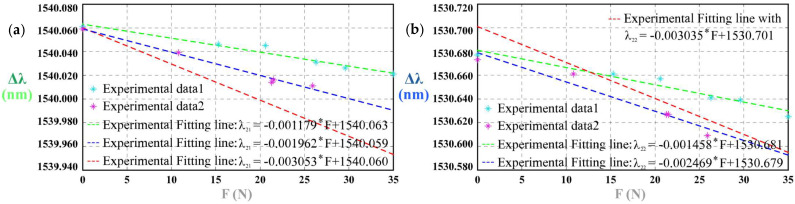
Comparison of the theoretical and the experimental sensitivity in Case II: (**a**) *λ*_21_ of 2^#^-II; (**b**) *λ*_22_ of 2^#^-II.

**Figure 11:**
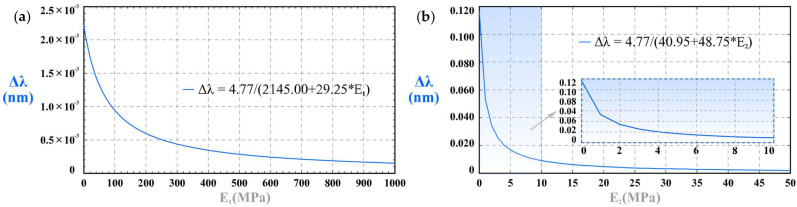
Effects of Young’s modulus on wavelength shifts in Case II: (**a**) plug-in sensor core’s Young’s modulus *E*_1_; (**b**) clip-on cap’s Young’s modulus *E*_2_.

According to Equation (20), the model predicts that the FBGs’ Bragg wavelength is a linear function of external force *F* and will be blue-shifted with the increase in F in Case II, which is in accord with the experimental results, shown in Figure 10. Figure 11a shows the relation of the wavelength shift Δ*λ* and the Young’s modulus of PSC *E*_1_ when the external force *F* = 50 N and *E*_2_ = 44 MPa. With the increase of *E*_1_, Δ*λ* reduces significantly; that is, the tactile sensitivity decreases sharply when *E*_1_ becomes greater at first. And then, when *E*_1_ becomes greater than 700 MPa, Δ*λ* gradually becomes unchanged and to be a constant. Figure 11b shows the effect of *E*_2_ on Δ*λ* when *F* = 50 N and *E*_1_ = 1.4 MPa. When *E*_2_ ≤ 5 MPa, Δ*λ* drops sharply with the increase of *E*_2_. After that, Δ*λ* tends to be unchanged with the change of *E*_2_. The change of *E*_2_ has a greater impact on tactile sensitivity than the change of *E*_1_.

## 5. Results and Discussion

The experimental results establish a clear trade-off between sensitivity and linearity across the two loading configurations. Case II (hollow PSC) delivers up to twice the force sensitivity observed in Case I (PSC with PSC-solid insert), whereas Case I maintains slightly higher linearity (R^2^ > 0.95). These trends are consistent with the parametric opto-mechanical model: increasing the Young’s modulus of either the plug-in sensor core (E_1_) or the clip-on cap (E_2_) reduces sensitivity, while employing a softer PSC-solid insert increases sensitivity. The direction of wavelength shift also accords with the model—red shift with increasing force in Case I and blue shift in Case II—further reinforcing the model’s explanatory power. Together with the proposed quality metric *Q* that balances sensitivity, linearity, and dynamic lag, the results indicate that softer solid inserts enhance tactile force perception while acknowledging the small loss in linearity relative to the hollow configuration.

Building on the above results—namely that Case II (hollow PSC) achieves up to twice the force sensitivity of Case I while Case I maintains slightly higher linearity (*R*^2^ > 0.95) and that the measurements agree with the geometry-aware closed-form model—we use the proposed quality metric *Q* to frame the sensitivity/linearity/lag balance. These findings provide a quantitative basis to articulate how the present clip-on cylindrical module addresses gaps highlighted in the Introduction (modularity and serviceability, form factor, and temperature compensation), alongside modeling and validation evidence (two configurations, model error < 8%). Guided by these axes, a concise comparison with prior FBG-based tactile sensors is presented in Table 6.

## 6. Conclusions

This work introduces a clip-on cylindrical FBG tactile module comprising a snap-fit Clip-on Cap (CC) and a plug-in Sensor Core (PSC), enabling rapid, plug-and-play reconfiguration. Two loading configurations were formulated and validated, and a closed-form, geometry-aware opto-mechanical model linked Bragg wavelength shifts to external force and module dimensions. Across configurations, model predictions remained within 8% of the experimental data. While current prototypes are constrained by a 2 Hz interrogator and by the cycle life of PolyJet elastomers, the combination of modular hardware, dual-case validation, and the *Q* metric offers a transferable framework for proposed deployable tactile force sensing. Future efforts will adapt this modular platform to additional sensing and actuation modules to broaden deployment across robotic and automated systems, and we will undertake additional studies to rigorously evaluate the sensor’s performance, robustness, and long-term stability across broader operating conditions.

## Figures and Tables

**Table 1:** Groups of tactile sensing experiment.

Group	Symbol	Details
Case I	1^#^-I-B	Responses to the impulse loads; a blue solid core inside
	1^#^-I-G	Responses to the impulse loads; a green solid core inside
	1^#^-I-LG	Responses to the impulse loads; a light green solid core inside
Case II	2^#^-II-1	Responses to the impulse loads; no solid core inside
	2^#^-II-2	Second responses to the impulse loads; no solid core inside

**Table 2:** Experiment results of 1^#^ PSC.

	Details	Sensitivity *K*_11-I_ (pm/N) *K*_12-I_ (pm/N)	Linear Correlation Coefficient *R*^2^_11_ *R*^2^_12_	Maximum Time Lag Δ*t*_11max_ (s) Δ*t*_12max_ (s)	*Q*—Factor *Q*_11_ (pm/N·s) *Q*_12_ (pm/N·s)
Experiment					
Blue		*K*_11-I-B_ = 0.5043	*R*^2^_11_ = 0.9848	Δ*t*_11max_ = 0.5	*Q*_11-B_ = 0.9932
		*K*_12-I-B_ = 0.5471	*R*^2^_12_ = 0.9566	Δ*t*_12max_ = 0.5	*Q*_12-B_ = 1.047
Green		*K*_11-I-G_ = 0.7047	*R*^2^_11_ = 0.9848	Δ*t*_11max_ = 0.5	*Q*_11-G_ = 1.3879
		*K*_12-I-G_ = 0.7221	*R*^2^_12_ = 0.9499	Δ*t*_12max_ = 0.5	*Q*_12-G_ = 1.3718
Light Green		*K*_11-I-LG_ = 1.407	*R*^2^_11_ = 0.9650	Δ*t*_11max_ = 1	*Q*_11-LG_ = 1.3578
		*K*_12-I-LG_ = 1.487	*R*^2^_12_ = 0.9520	Δ*t*_12max_ = 1	*Q*_12-LG_ = 1.4156

**Table 3:** Experiment results of 2# PSC.

	Details	Sensitivity *K*_21-II_ (pm/N) *K*_22-II_ (pm/N)	Linear Correlation Coefficient *R*^2^_21_ *R*^2^_22_	Maximum Time Lag Δ*t*_21max_ (s) Δ*t*_22max_ (s)	*Q*—Factor *Q*_21_ (pm/N·s) *Q*_22_ (pm/N·s)
Experiment					
First		*K*_2_1-__II__-__1__ = −1.179	*R*^2^_2_1__ = 0.955	Δ*t*_2_1max__ = 1	*Q*_21_ = 1.1259
		*K*_2_2-__II__-__1__ = −1.458	*R*^2^_2_2__ = 0.949	Δ*t*_2_2max__ = 1	*Q*_22_ = 1.3836
Second		*K*_2_1-__II__-__2__ = −1.962	*R*^2^_2_1__ = 0.988	Δ*t*_2_1max__ = 0.5	*Q*_21_ = 3.8769
		*K*_2_2-__II__-__2__ = −2.469	*R*^2^_2_2__ = 0.947	Δ*t*_2_2max__ = 1	*Q*_22_ = 2.3381

**Table 4:** Lists of symbols.

Symbol	Description	Symbol	Description
*E*_0_	Young’s modulus of FBG	*r*_0_	Radius of FBG
*F*	External concentrated force	*F*_10_	Load on the top grooved block of the spherical cap of the clip-on cap
*F*′_10_	Load on the top of the PSC-solid	*E*_1_	Young’s modulus of the plug-in sensor core
*A*_1_	Cylindrical cross-sectional area of the plug-in sensor core	*r*_λ_	Center distance of the through-hole
*r*_1_	Outer radius of the plug-in sensor core	*r*′	Radius of alignment pillar on the plug-in sensor core
*r*_10_	Inner radius of the plug-in sensor core	*E*_2_	Young’s modulus of the clip-on cap
*A*_2_	Cylindrical cross-sectional area of the clip-on cap	*r*_2_	Outer radius of the clip-on cap
*r*_20_	Inner radius of the clip-on cap	*E*_S_	Young’s modulus of the PSC-solid
*A*_s_	Cross-sectional area of the PSC-solid		

**Table 5:** Properties of the clip-on tactile sensor module.

	Type	Case I	Case II
Parameters			
*λ* (nm)		*λ*_11_ = 1546.295	λ_21_ = 1540.060
		*λ*_12_ = 1524.570	λ_22_ = 1530.701
*p*_e_		0.22	0.22
*r*_0_ (μm)		62.5	62.5
*r*_10_ (mm)		9	9
*r*_1_ (mm)		10.5	10.5
*r*_λ_ (mm)		12	12
*r*_20_ (mm)		15.5	15.5
*r*_2_ (mm)		17	17
*E*_0_ (Pa)		7.4 × 10^10^	7.4 × 10^10^
*E*_1_ (Pa)		3.2 × 10^6^	1.4 × 10^6^
*E*_2_ (Pa)		44 × 10^6^	44 × 10^6^
*E*_s_ (Pa)		*E*_S-B_ = 2.0 × 10^9^	—
		*E*_S-G_ = 7.2 × 10^8^	—
		*E*_S-LG_ = 3.0 × 10^7^	—

**Table 6:** Contributions of the present work relative to prior FBG-based tactile sensors.

Aspect	Prior FBG-Based Tactile Sensors	This Work
Modularity & serviceability	Laminated skins; hot-swap rare [21,23,25]	Clip-on two-piece (CC + PSC); swap in seconds.
Form factor focus	Planar/dome/palm; cylindrical tip modules uncommon [21,22,23,25]	Cylindrical, tip-mounted
Temperature compensation	Reference FBG often omitted [22,25]	Integrated Temp-FBG in PSC
Scalable, closed-form modeling	No cross-geometry closed form [20,21,23]	Geometry-aware closed form; scriptable sizing 10–22 mm
Configurations & validation	—	Two cases (PSC-solid/hollow PSC); model error < 8%
Performance trade-off metric	—	Quality metric Q balances sensitivity, linearity, and lag
Quantified performance outcomes	—	Case II sensitivity ≈ 2 × Case I; Case I *R*^2^ > 0.95

**Figure A4:**
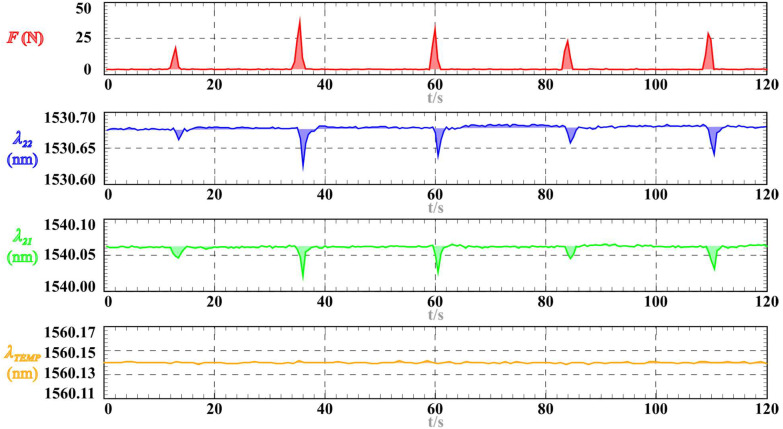
Bragg wavelength shift (Δ*λ*) versus applied force (F)—Case II.

**Figure A5:**
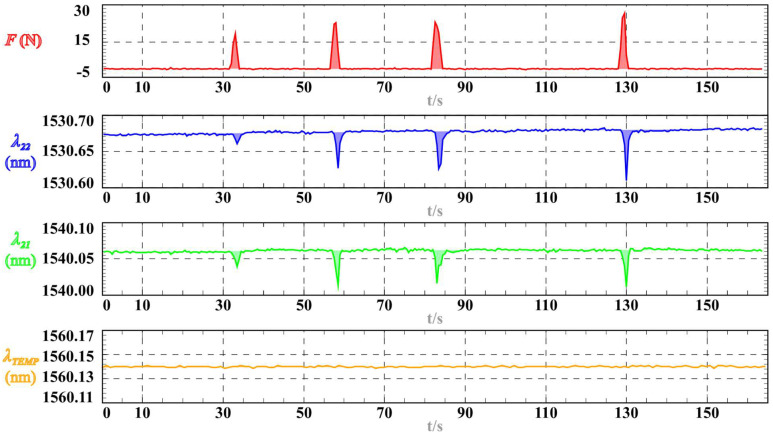
Bragg wavelength shift (Δ*λ*) versus applied force (F)—Case II.

## Data Availability

The datasets generated during this study are available from the corresponding author on reasonable request.

## Abbreviations

The following abbreviations are used in this manuscript:

ABS	Acrylonitrile Butadiene Styrene
CC	Clip-on Cap
EMI	Electromagnetic Interference
FBG	Fiber Bragg Grating
LED	Light-Emitting Diode
PSC	Plug-in Sensor Core
PSC-solid	Plug-in Solid Core
Temp-FBG	Temperature-Reference FBG
UV	Ultraviolet
VTSs	Vision-based Tactile Sensors
SMF	Single-Mode Fiber

## Appendix A

This appendix presents standalone figures referenced in the main text. All axes, units, and symbols follow the same conventions as in the manuscript. Unless otherwise noted, the experimental conditions and data-processing procedures are identical to those described in the corresponding sections.
